# A novel form of necrosis, TRIAD, occurs in human Huntington’s disease

**DOI:** 10.1186/s40478-017-0420-1

**Published:** 2017-03-08

**Authors:** Emiko Yamanishi, Kazuko Hasegawa, Kyota Fujita, Shizuko Ichinose, Saburo Yagishita, Miho Murata, Kazuhiko Tagawa, Takumi Akashi, Yoshinobu Eishi, Hitoshi Okazawa

**Affiliations:** 1 Department of Neuropathology, Medical Research Institute, Tokyo Medical and Dental University, 1-5-45, Yushima, Bunkyo-ku, Tokyo, 113-8510 Japan; 2 Department of Neurology, National Hospital Organization, Sagamihara National Hospital, 18-1, Sakura-dai, Minami-ku, Yokosuka, 252-0392 Japan; 3 Research Center for Medical and Dental Sciences, Tokyo Medical and Dental University, 1-5-45, Yushima, Bunkyo-ku, Tokyo, 113-8510 Japan; 4 Department of Neurology, National Center of Neurology and Psychiatry, 4-1-1, Ogawahigashimachi, Kodaira, Tokyo, 187-8551 Japan; 5 Department of Human Pathology, Medical Research Institute, Tokyo Medical and Dental University, 1-5-45, Yushima, Bunkyo-ku, Tokyo, 113-8510 Japan; 6 Department of Neuropathology, Center for Brain Integration Research, Tokyo Medical and Dental University, 1-5-45, Yushima, Bunkyo-ku, Tokyo, 113-8510 Japan

**Keywords:** TRIAD, Necrosis, Huntington’s disease, LATS, Neurodegeneration, TEAD, YAP, Hippo pathway, Electron microscopy

## Abstract

We previously reported transcriptional repression-induced atypical cell death of neuron (TRIAD), a new type of necrosis that is mainly regulated by Hippo pathway signaling and distinct from necroptosis regulated by RIP1/3 pathway. Here, we examined the ultrastructural and biochemical features of neuronal cell death in the brains of human HD patients in parallel with the similar analyses using mutant Htt-knock-in (Htt-KI) mice. LATS1 kinase, the critical regulator and marker of TRIAD, is actually activated in cortical neurons of postmortem human HD and of Htt-KI mouse brains, while apoptosis promoter kinase Plk1 was inactivated in human HD brains. Expression levels of YAP/YAPdeltaC were decreased in cortical neurons of human HD brains. Ultra-structural analyses revealed extreme enlargement of endoplasmic reticulum (ER), which characterizes TRIAD, in cortical neurons of human HD and those of Htt-KI mice. These biochemical and morphological results support that TRIAD occurs in human and mouse neurons under the HD pathology.

**Electronic supplementary material:**

The online version of this article (doi:10.1186/s40478-017-0420-1) contains supplementary material, which is available to authorized users.

## Introduction

The nature of cell death in neurodegenerative diseases remains obscure. A number of clinical trials against neurodegenerative diseases using anti-apoptosis or anti-necroptosis chemicals were so far unsuccessful. The therapeutic effect of rapamycin on mouse model of amyotrophic lateral sclerosis (ALS) is controversial [16, 18]. Minomycin, which has anti-apoptosis and anti-inflammatory effects and whose therapeutic effect on ALS mouse model was reported [19], caused notable deterioration instead of amelioration in human clinical trial of ALS patients [2]. Though necrosis, apoptosis or autophagic cell death has been implicated in neurodegeneration, actual phenotype of neuronal death in vivo, actual molecular mechanisms to explain the cell death in vivo, and relative contribution of different prototypes of cell death to neurodegeneration in vivo are still largely unknown.

We proposed previously that the atypical necrosis induced by transcriptional repression (TRIAD) defined by extremely enlarged and unstable ER with intact mitochondria and nuclei, could be a prototype of cell death in the HD pathology [3]. The necrotic cell death (or Type III cell death in the category by Schweichel and Merker) [15] was induced by the RNA-polymerase inhibitor, α-amanitin, and suppressed by new isoforms of YAP [3, 8] that interacts with transcription factor TEAD or p73 as a critical mediator of Hippo signaling pathway.

The molecules involved in TRIAD were comprehensively analyzed using Drosophila genetic screen, and the identified genes were integrated to the network executing TRIAD [9]. The analysis newly identified that splicing disturbance caused by decreased expression of multiple hnRNPs additively enhanced TRIAD [9]. Moreover, we revealed that mutant Htt-Exon1 expression at a physiological level induces TRIAD in primary cortical neurons and that targeting of TEAD/YAP-dependent TRIAD recovers HD mouse models [8].

In the previous works, we also revealed that two kinases, LATS and Plk1, switch apoptosis and necrosis (TEAD/YAP-dependent necrosis TRIAD) in neurons through the balance of cytoplasmic and nuclear YAP and the switch of transcription factors interacting with YAP [8]. Activation of Plk1 increases the ratio of apoptosis in relevance to necrosis, while activation of LATS increases the ratio of necrosis but suppresses apoptosis in neurons in which proliferative cell-specific Plk1 is usually inactive [8]. In this scheme, single activation of LATS without Plk1 more strongly promotes necrotic cell death TRIAD [8].

However, the questions remain on how activities of these kinases are actually changed in vivo in human HD and whether TRIAD-specific morphological changes actually occur in vivo in human HD brains. Although we previously reported aberrant expression of YAPdeltaC in motor neurons of ALS model mice [12] while we could not directly indicate the existence of TRIAD by ultra-structural analysis of the cell death or by new markers of TRIAD such as LATS1 and Plk1 that had been reported later.

In this study we employed these new tools and addressed whether TRIAD occurs in the brains of human HD patients and mutant Htt-KI mice. The results obviously supported that TRIAD actually occurs in human HD brains.

## Materials and methods

### HD model mice

Mutant Htt-KI mice are a generous gift from Prof. Marcy MacDonald (Massachusetts General Hospital, Harvard Medical School) [17] in which human mutant Htt carrying 111CAG repeats is integrated. Their original genetic background was 129SvEv/CD1 (mixed background by crossing 129SvEv male and CD1 female) [17]. However, their genetic background had been changed to C57BL/6 when we received mutant Htt-KI mice. Furthermore, we crossed the male mutant Htt-KI mice with female C57BL/6 mice for more than 5 generations before this study. Accordingly, C57BL/6 mice were used as negative controls in this study.

### Human brains

We obtained informed consent and ethics committee approval (Sagamihara National Hospital, NCNP and TMDU) to examine autopsy specimens from three HD patients and three control patients without neurological disorders (lung cancer, leukemia, and cholangiocarcinoma). The diagnosis of HD was confirmed by genetic analysis of CAG repeat of Htt gene. Frontal cortex from five HD patients and two PSP patients were used for ultra-structural analyses. Frontal and parietal cortex tissues of three HD patients and five non-neurological disease patients were used for immunohistochemistry.

### Western blotting

Brain tissues were dissected from Htt-KI mice or littermate control mice and washed three times with ice-cold PBS and dissolved in lysis buffer containing 62.5 mM Tris–HCl (pH 8.0), 2% (w/v) SDS, 2.5% (v/v) 2-mercaptoethanol and 5% (v/v) glycerol. The protein concentration was quantified using the BCA method (Micro BCA Protein Assay Reagent Kit, Thermo Fisher Scientific, MA, USA). Primary and secondary antibodies were diluted for immunoblotting as follows: rabbit anti-LATS1 (1:2000, Cell Signaling Technology, MA, USA, #3477), rabbit anti-phospho-LATS1 (Ser909, 1:5000, Cell Signaling Technology, MA, USA, #9157), mouse anti-PLK1 (1:2000, Invitrogen, MA, USA, #37-7000), anti-phospho-PLK1 (Thr210, 1:30000, Abcam, Cambridge, UK, #ab155095); HRP-conjugated anti-mouse IgG (NA931VA) and anti-rabbit IgG (NA934VS) (both of them, 1:3000, GE Healthcare, Buckinghamshire, UK). Antibodies were diluted in Can Get Signal (TOYOBO, Osaka, Japan). ECL prime (GE Healthcare, Buckinghamshire, UK) was used to detect the bands using LAS4000 (GE Healthcare, Buckinghamshire, UK) [8].

### Immunohistochemistry

Immunohistochemistry was performed as previously described with minor modifications [8]. After deparaffinization, rehydration, and antigen retrieval (microwaved in 10 mM citrate buffer, pH 6.0, at 100 °C, 5 min, three times), the sections were incubated sequentially with 0.5% TritonX-100 in PBS for 30 min at room temperature (RT) to membrane permeation, with 10% FBS for 60 min at RT, with primary antibodies: rabbit anti-phospho-LATS1 (Ser909, 1:100, Cell Signaling Technology, MA, USA, #9157), rabbit anti-phospho-PLK1 (Thr210, 1:100, Abcam, Cambridge, UK, #ab155095), mouse anti-MAP2 (1:200, Santa Cruz, TX, USA, #sc-32791) and mouse anti-NeuN (1:100, Abcam, Cambridge, UK, ab104224) one or two overnight, and finally with secondary antibodies: Alexa Flour 488-labeled anti-mouse IgG (1:1000, Invitrogen, MA, USA) and Cy3-labeled anti-rabbit IgG (1:500, Jackson ImmunoResearch, PA, USA) for 1 h at RT. Images were acquired by confocal microscopy: Olympus FV1200 (Olympus, Tokyo, Japan) and LSM710 (Carl Zeiss, Oberkochen, Germany).

### Signal acquisition from immunohistochemistry

Immunohistochemistry images obtained by Olympus FV1200 were next analyzed by Image-J (NIH, MD USA: https://imagej.nih.gov/ij/). Signal intensities (AU/pixel) of YAP, YAPdeltaC, phospho-LATS1 and phospho-PLK1 in each neuron (NeuN-positive or MAP2-positive cell) were quantified by free-hand-surrounding the shape of neuron with Image-J. From human immunohistochemistry images 4 visual fields were randomly selected, and 100 neurons in total were analyzed. Background signals were collected from 8 areas that did not include cells, and the signal intensity of each neuron was subtracted with the mean value of the background signals. The mean value of the signal intensities of 100 neurons after subtraction of the background signals was used as the representative value for a patient or a control, and statistical analysis was performed between 3 patients and 3 controls.

### Electron microscopy

Electron microscopic observation was performed basically following the method described previously [8]. After deparaffinization and rehydration, tissues were washed with PBS three time, fixed in 2.5% glutaraldehyde/0.1 M phosphate buffer (PB) (pH7.4), and treated with 1% OsO_4_/0.1 M PB for 2 h. Fixed tissues were dehydrated through a graded ethanol series and embedded in epoxyresin. Ultrathin sections were stained with uranyl acetate and lead citrate. Data acquisition was performed with a transmission electron microscope (H-9000, H7600 or H-7100, Hitachi, Tokyo, Japan).

Regarding human brain samples, frontal cortex were fixed in 2.5% glutaraldehyde/0.1 M cacodylate buffer for 2 h and treated with 1% OsO_4_/0.1 M cacodylate buffer between 90 min and 2 h, within 5 h after death.

### Statistical analysis

Statistical analyses were performed with Student’s *t*-test.

## Results

### Activation of LATS1 and Plk1 in Htt-KI mice

**Fig. 1:**
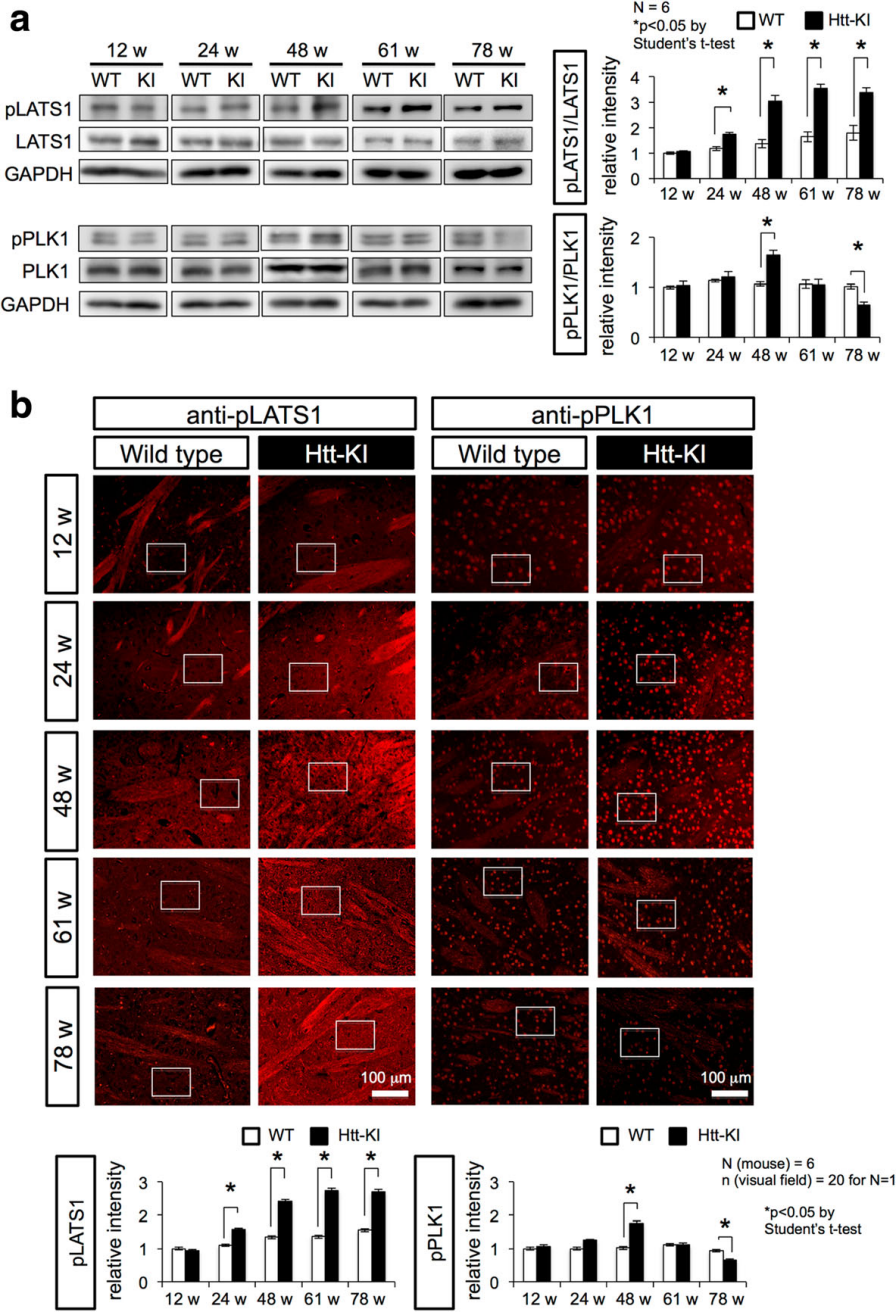
LATS1 and PLK1 are activated in Htt-KI mice. **a** Chronological analyses of LATS1, PLK1 and their phosphorylated forms by western blot with cerebral tissues including striatum from Htt-KI and their background mice (WT). The right graphs show the ratios of pLATS1/LATS1 and pPLK1/PLK1 in western blot analyses that are corrected by the value of WT at 12 weeks. **b** Immunostaining of striatal tissues from mutant Htt-KI (111Q) and their background mice with anti-phospho-LATS1 and anti-phospho-PLK1 antibodies. The lower graphs show relative intensities of immunostains in squared areas after subtraction with background stain signals. Mean value of signal intensity/area from twenty visual fields was used for *N* = 1. The bar graphs show mean + SEM. *: *p* < 0.05 in Student’s *t*-test

We tested whether LATS1 and Plk1 are activated in vivo during the aging of mutant Htt-knock-in (KI) mice. Western blots revealed that activated forms of LATS1 (phospho-LATS1) was increased in comparison to total LATS1 from 6 months during the progression of pathology in the striatum of mutant Htt-KI mice in vivo (Fig. 1a). Phospho-Plk1 was also increased but only at 48 weeks of age (Fig. 1a). The increase of phospho-Plk1 was transient and it was decreased at later time points (Fig. 1a). Consistently, immunohistochemistry revealed that signal intensities of phospho-LATS1 and phospho-Plk1 in the striatum of mutant Htt-KI mice were increased during the progression of pathology from 12 to 48 weeks of age (Fig. 1b). The signal intensities of phospho-LATS1 stayed at high levels while the signals of phospho-Plk1 became weaker at later stages (Fig. 1b).

**Fig. 2:**
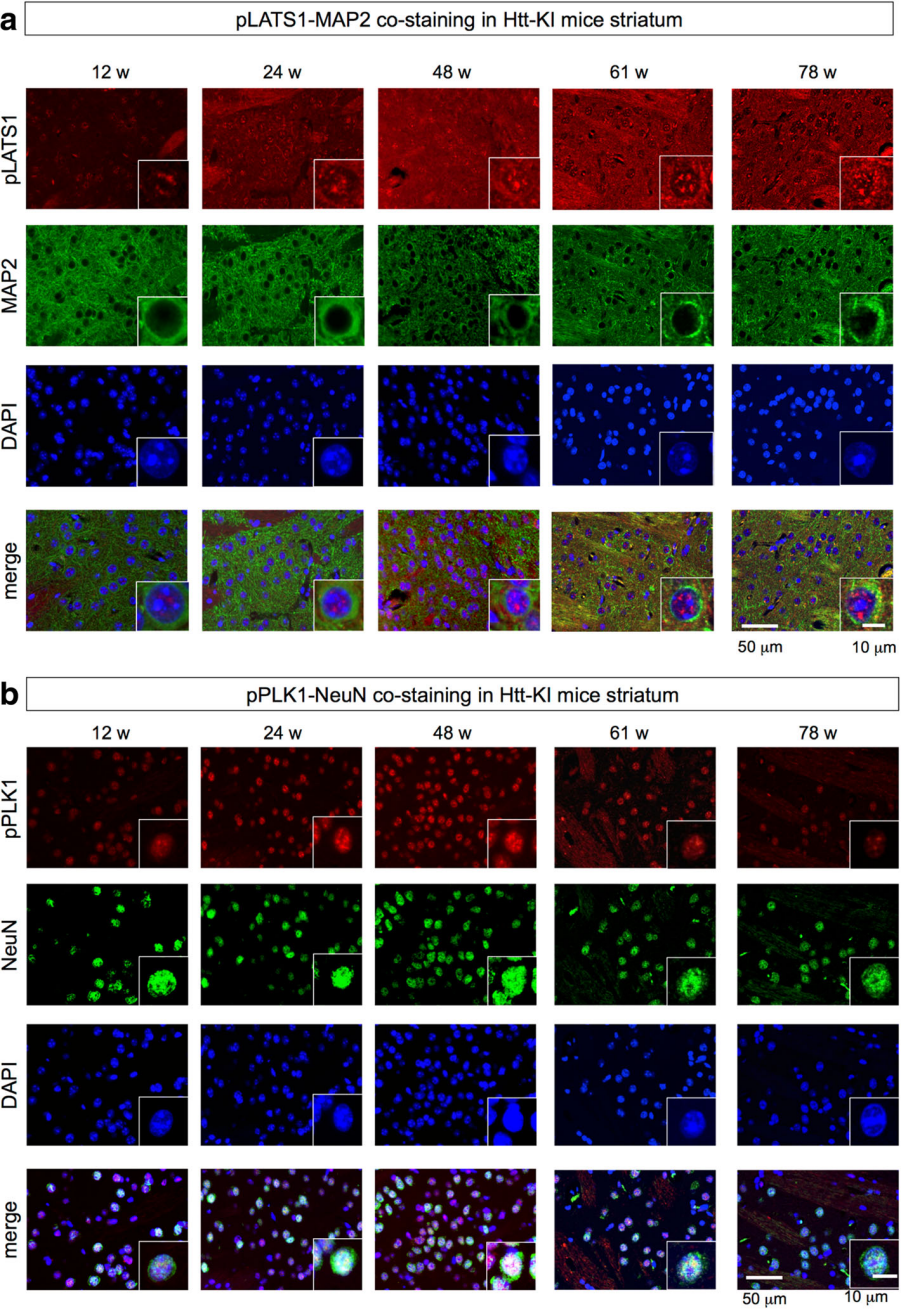
LATS1 and Plk1 are activated in striatal neurons of Htt-KI mice. **a** Double staining of LATS1 and MAP2 revealed LATS1 activation in neurons of mutant Htt-KI mice. LATS is expressed mainly in the nuclei rather than the cytoplasm of neurons. **b** Double staining of PLK1 and NeuN revealed transient actication in neurons of mutant Htt-KI mice. Plk1 is expressed in neurons

Immunohistochemistry with neuronal markers, MAP2 and NeuN confirmed that the cells with activated LATS1 or Plk1 were actually striatal neurons of mutant Htt-KI mice (Fig. 2a, b). These results support that the biochemical condition where TRIAD can execute [8], i.e. the condition where single activation of LATS or dual activation of two kinase, exists in striatal and cortical neurons at the late stage of mutant Htt-KI mice.

### Activation of LATS1 in human HD brains

**Fig. 3:**
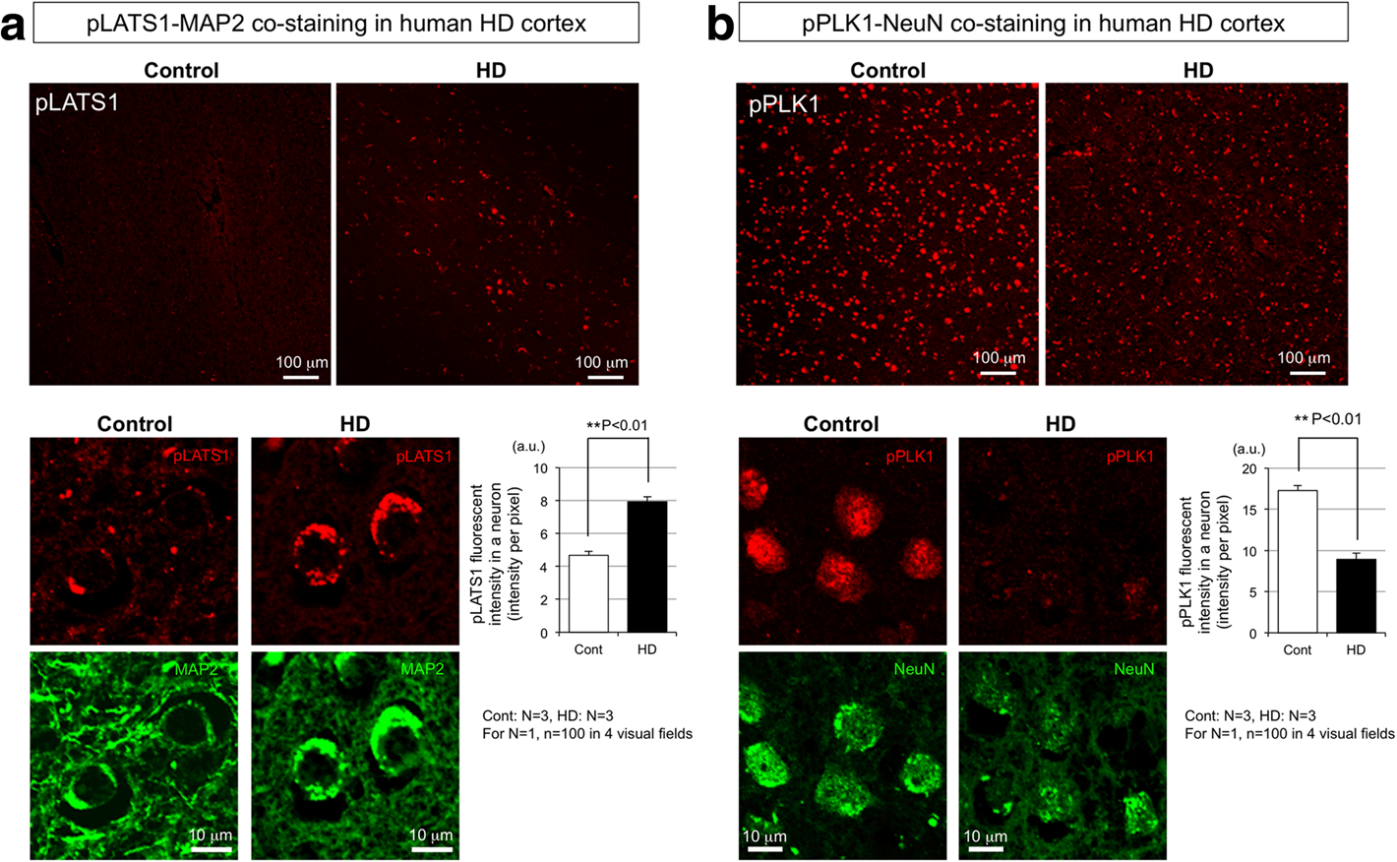
LATS1 is activated in cortical neurons of postmortem human HD patients. **a** Co-staining of phosphorylated LATS1 and MAP2 with cerebral cortex (frontal cortex) of HD patients (grade IV) and non-neurological disease control. At low magnification, signal intensities were high in HD but low in the control (*upper panel*, *lower graph*). High magnification revealed that the signals were localized to MAP2-positive neurons (*lower panels*). The lower graph shows mean + SEM of the pLATS1 signal intensity per neuron of three patients or three controls (for *N* = 1, mean value of signal intensities of 100 neurons from 4 visual fields were used). Non-neurological disease patients were used as control. **: *p* < 0.01 in Student’s *t*-test. **b** Co-staining of phosphorylated PLK1 and NeuN with cerebral cortex (*frontal cortex*) of HD patients (grade IV) and non-neurological disease control. At low magnification, signal intensities were high both in HD and control (*upper panel*, *lower graph*). High magnification revealed that the signals were localized to NeuN-positive neurons (*lower panels*). The lower graph shows mean + SEM of the PLK1 signal intensity per neuron of three patients or three controls (for *N* = 1, mean value of signal intensities of 100 neurons from 4 visual fields were used). Non-neurological disease patients were used as control. **: *p* < 0.01 in Student’s *t*-test

The immunohistochemistry was performed similarly with human HD brains to examine LATS1 and Plk1 activation (Fig. 3a, b). HD patients were diagnosed clinically and genetically with CAG repeat expansion. We found phospho-LATS1 in cortical neurons was increased in HD than control (Fig. 3a, upper panels, lower panels, lower graph). The pattern of phosphorylated LATS1 stains in neurons was homologous to that in mutant Htt-KI mice (Fig. 2a).

On the other hand, activation of Plk1, which induces apoptosis rather than TRIAD [8], was not obvious. At low magnification, the basal level of phosphorylated Plk1 was already high in the control, and it was almost similar or slight lower in HD (Fig. 3b, upper panel). At high magnification, phospho-Plk1 stains were found in the nucleus of a part of neurons (Fig. 3b, lower panel), while the intensity in the cytoplasm and the number of stain positive neurons were decreased (Fig. 3b, lower graph). The decrease of phospho-Plk1 in human cortical neurons might be consistent with the late-stage reduction of phospho-Plk1 in striatal neurons of mutant Htt-KI mice. The morphology of Plk1-positive neurons in the control brains was normal at least at the level of fluorescent microscopy.

### Decrease of YAP/YAPdeltaC in human HD brains

**Fig. 4:**
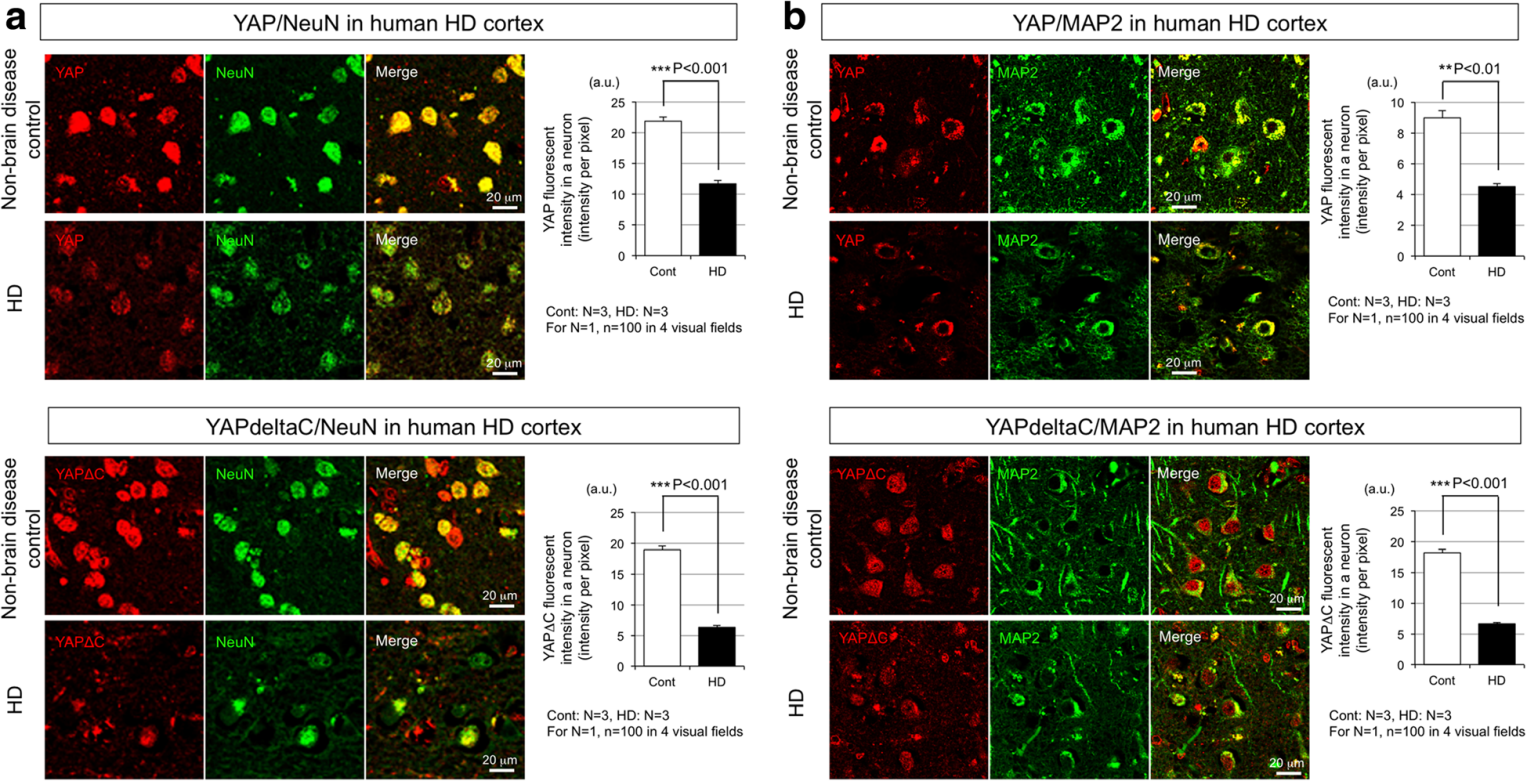
YAP/YAPdeltaC are decreased in cortical neurons of postmortem human HD patients. **a** YAP (*upper panels*) and YAPdeltaC (*lower panels*) staining in NeuN-positive neurons in the cerebral cortex of HD and non-neurological disease control patients. Both of the YAP and YAPdeltaC signals were decreased in HD than control. The right graphs show mean + SEM of YAP/YAPdeltaC signal in a neuron (mean intensity/pixel). ***: *p* < 0.001 in Student’s *t*-test. **b** YAP (*upper panels*) and YAPdeltaC (*lower panels*) staining in MAP2-positive neurons in the cerebral cortex of HD and non-neurological disease control patients. Both of the YAP and YAPdeltaC signals were decreased in HD than control. The right graphs show mean + SEM of YAP/YAPdeltaC signal in a neuron (mean intensity/pixel). **: *p* < 0.01, ***: *p* < 0.001 in Student’s *t*-test

Our previous analysis revealed decreased YAP/YAPdeltaC protein expression in the nucleus of Htt-KI [8]. Consistently, YAP and YAPdeltaC were decreased in the nuclei of neurons in the cerebral cortex of human HD patients (Fig. 4a, b). The results further supported that necessary conditions for TRIAD exist in human cortical neurons under the HD pathology. As mentioned, striatal neurons were hardly detected in human HD brains, thus cortical neurons in frontal and parietal cortex were examined for the analysis of YAP and YAPdeltaC.

### TRIAD occurs in mouse HD model brains

**Fig. 5:**
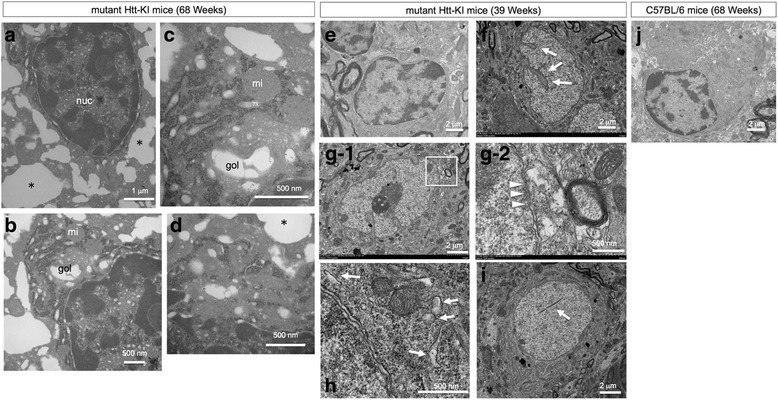
TRIAD-specific changes in cortical neurons of Htt-KI mice. **a** Electron microscopy revealed numerous cytoplasmic vacuoles (*asterisk*) despite of normal nucleus (nuc). **b** Golgi apparatus was also enlarged, while mitochondria (mi) did not expand and the mitochondrial network structure remained. **c** A larger magnification of mitochondria and Golgi apparatus. **d** No subcellular organ was involved in the vacuole (*asterisk*) while ribosome was not confirmed throughout the cell. No autophagic vacuoles were observed at a high magnification. The image was identical to the change of TRIAD in primary cortical neurons, in which vacuoles turned out to be ER [3]. **e** A large part of neurons at 39 weeks seemed normal, while the space between inner and outer nuclear membranes were slightly dilated. **f** In 20–30% of neurons, invagination of nuclear membrane was observed (*arrow*). **g**-1 In such abnormal cells, loss of our nuclear membrane (*arrowhead*) and cytoplasmic vacuoles (*squared area*) were frequently observed. **g**-2 Larger magnification of the squared area is shown. **h** Mild ER dilatation was also observed in other cells without nuclear invagination. **i** A few neurons possessed intranuclear fibrils corresponding to nuclear inclusion body. **j** In the background mice (C57BL/6) at 68 weeks, neurons show no TRIAD-related morphological changes

Ultra-structural analyses of cortical neurons in Htt-KI mice at 68 weeks by electron microscopy revealed that a number of neurons possessed large cytoplasmic vacuoles, while their nuclei seemed largely normal (Fig. 5a). The ratio of electron-dense hetrochromatin was increased slightly but the feature was obviously different from chromatin condensation (Fig. 5a). Apoptotic body was not detected (Fig. 5a). Larger magnification revealed detachment of ribosomes from ER and their clustering in the cytoplasm (Fig. 5b, c). ER lumen was enlarged and the enlargement seemed to be developed to cytoplasmic vacuoles in some cases (Fig. 5b, c). Inner and outer nuclear membranes were separated at some portions of the nucleus (Fig. 5b). Golgi apparatus was also expanded (Fig. 5b, c). Such intracellular vacuoles did not contain organelle, and definite autophagosome or autolysosome was not detected (Fig. 5d). Apoptotic changes were also not observed. These features were largely consistent with our previous observation of primary neurons under TRIAD induced by alpha-amanitin [3].

Such morphological changes were not dominant in ultra-structural analyses of cortical neurons in Htt-KI mice at 39 weeks before the onset (the onset is around 50 weeks) (Fig. 5e). However, we found that some neurons showed mild dilatation of ER, loss of our nuclear membrane connected to ER and/or nuclear membrane invagination (Fig. 5f–h), which might suggest initiation of morphological change of TRIAD. No definite nuclear chromatin condensation or fragmentation was observed. In addition, we found that a few neurons possessed intranuclear fibrils that might correspond to intranuclear Htt inclusion bodies (Fig. 5i). In the background mice (C57BL/6) at 68 weeks, we found no such TRIAD-related morphological changes in the cortex and the striatum (Fig. 5j).

### TRIAD occurs in human HD brains

**Fig. 6:**
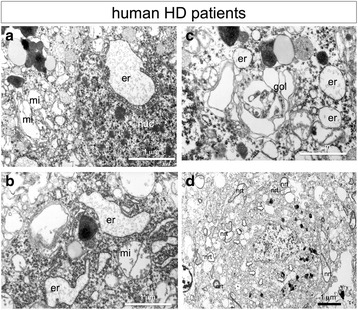
TRIAD-specific changes in cortical neurons of human HD patients. **a** ERs (er) were extremely enlarged in cortical neurons of a HD patient (grade III). Mitochondria were also enlarged (mi). **b** Ribosomes still attach to the outer surface of enlarged ER (er), and could be discriminated from mitochondria (mi). **c** Golgi apparatus (gol) was also enlarged. **d** At a lower magnification, a number of ER was enlarged while the nucleus was almost normal. Autophagosomes were not detected in the cell. Neurite is indicated as nrt. EM images of other three patients (grade 4) are shown in Additional file 1: Figure S1

We further performed ultra-structural analysis to human HD patient brains (Fig. 6, Additional file 1: Figure S1). Four HD patients that had been diagnosed by autosomal dominant familial history, clinical manifestation such as chorea and dementia, and CAG repeat expansion more than 40 were used for the analysis. One of them was at grade III and three of them were at grade IV. Electron microscopy revealed enlarged ER in cortical neurons (Fig. 6a, er). The nucleus was almost normal and did not show chromatin condensation (Fig. 6a, nuc). Mitochondria were also expanded (Fig. 6a, mi), suggesting the late stage of TRIAD. Mitochondria and ER were discriminated easily by size and shape of the content granules, by ribosomes attached to ER membrane, and by the remaining cristae of mitochondria (Fig. 6b, er and mi). Golgi apparatus was sometimes expanded (Fig. 6c, gol). In a cell surrounded by neurites (dendrites or axons) (Fig. 6d, nrt), a number of enlarged ER was found (Fig. 6d, white arrow) while autophagosome was hardly detected. No definite features of apoptosis were observed in neurons of HD patients. Neurons in the striatum were extremely few or none in the autopsy HD brains (grade III and IV) and their morphological analysis was impossible.

We also analyzed two cases of progressive supranuclear palsy (PSP) as disease controls. The fixation of brain tissues for ultrastructural analysis was completed within 5 h after death of the HD and PSP patients. On the other hand we could not find brain samples of non-neurological disease patients in bio-resource of our hospitals or in the other brain banks in Japan that could be used as control. Unexpectedly, the expansion of ER was also detected in PSP although the extent of ER expansion was less prominent than in HD patients (Additional file 1: Figure S1, Additional file 2: Figure S2 and Additional file 4: Table S1). Lipofuscin granules were frequently observed in neurons of HD and PSP patients.

## Discussion

In our previous studies, we proposed TRIAD as an atypical cell death that can be categorized to Type 3 necrotic cell death and might be relevant to the cell death in neurodegenerative diseases [3]. Simultaneously, we identified YAP isoforms (full-length YAP2 and YAPdeltaC) as molecules regulating TRIAD [3]. We next analyzed the feature of cell death in the cell model of HD, and found that overexpression of Htt-Exon1 induced Type 3 necrotic cell death that is morphologically and biochemically identical to TRIAD [8]. Moreover, we revealed that ER instability was enhanced in living neurons of two HD model mice (mutant Htt-Exon1-transgenic R6/2 mice [7] and mutant Htt-knock-in mice [17]) and showed that targeting the TEAD/YAP-mediated transcription or the Hippo pathway ameliorated the cell death and symptoms of HD model mice [8].

However, the evidences were insufficient to prove that the new type of necrosis actually occurs in human HD pathology. To address the question, we directly investigated cerebral neurons in human HD. Consequently we confirmed activation of the TRIAD-linked kinase LATS1 [8], inactivation of apoptosis promoting kinase Plk1 [8], and ultra-structural changes of ER actually in human HD brains that was morphologically identical to TRIAD.

**Fig. 7:**
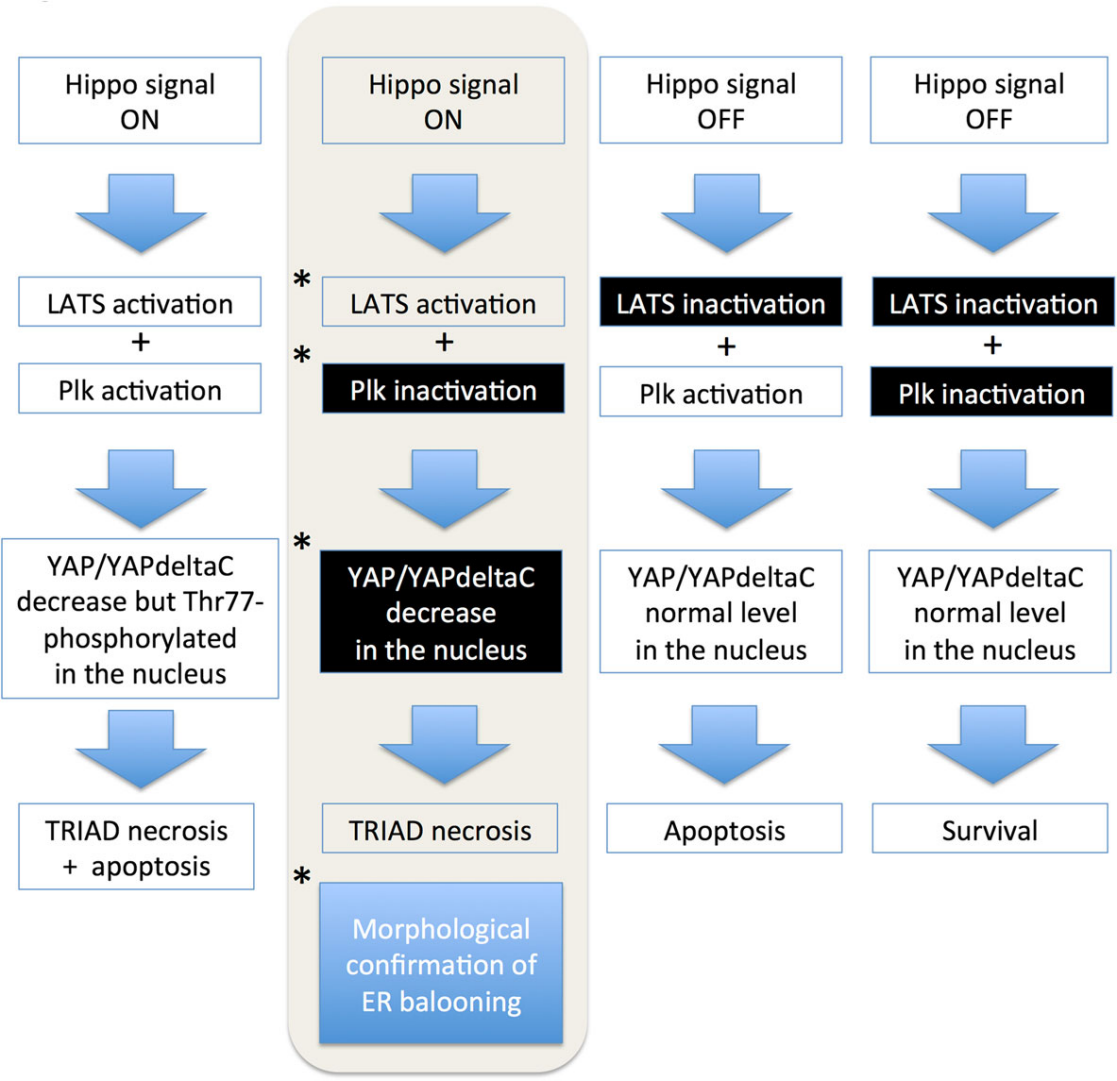
Signaling pathway and conditions for TRIAD are consistent for the observation in human HD neurons. The second panel from the left shows signaling pathway and condition necessary for TRIAD. Our observation in human HD brains supports these conditions are satisfied in cortical neurons of human HD. In addition, our analyses indicated the morphological changes consistent with TRIAD. Plk1 activation shifts the partner transcription factor of YAP/YAPdeltaC from TEAD to p73, thereby shifting the signal from survival to apoptosis [8]. The loss of YAP/YAPdeltaC-TEAD survival signal leads to TRIAD type of necrosis [8]. Asterisk indicates the condition confirmed in human HD neurons by this study

In more details, we found the transient activation and the later suppression of Plk1 in neurons of mutant Htt-KI mice (Fig. 1). Plk1 was suppressed in human postmortem HD brains (Fig. 3b). Plk1 strongly enhances apoptosis by increasing YAP-p73 interaction [8]. In addition, Plk1 weakly promotes TRIAD necrosis by partially suppressing YAP-TEAD interaction [8]. Therefore, Plk1 inhibitor ameliorates TRIAD although weakly [9]. Intriguingly, the contribution of LATS1 to TRIAD is larger than that of Plk1 [8]. Biochemical and morphological changes are highly consistent in mouse and human HD brains assuming that the end-point pathology in human postmortem brains reflects the late stages of mouse HD model (Additional file 3: Figure S3). Considering with the previously identified signaling and conditions necessary and sufficient for TRIAD [8], these results indicated that TRIAD occurs in neurons under the human HD pathology (Fig. 7).

In EM analysis of human HD brains, we found mitochondrial enlargement (Additional file 4: Table S1) that had been unusual at the early stage of TRIAD in primary neurons [3]. This finding might suggest the possibility that TRIAD partially shares apoptotic signaling in vivo. Our previous screening by fly model that revealed partial share of signaling molecules in TRIAD and apoptosis, might support this idea [9]. Given that Plk1 was reported essential for recovering mitochondrial dysfunction [10], the decrease of Plk1 at the late stage of human and mouse HD pathologies might affect mitochondrial integrity and induce morphological changes of mitochondria in human postmortem HD brains. However, detailed mechanisms underlying the mitochondrial changes in TRIAD need further investigation. Especially, it would be of significance to analyze chronologically YAP phosphorylation at Thr77 and Ser127 that shifts the balance between apoptosis and TRIAD necrosis [8], in parallel with YAP phosphorylation at Tyr357 by c-Abl, a DNA damage signal mediator, that switches on/off apoptosis [4]. These analyses might elucidate factors that modify the TRIAD prototype in vivo and in human, and should be performed in the future.

Interestingly, necrotic cell death such as “ballooned neuron” or “neuronal achromasia” has been described in tauopathy like corticobasal degeneration [1, 13], Pick’s disease (a form of frontotemporal dementia) [5] and progressive supranuclear palsy (PSP) [11]. As achromasia is based on ER staining, it is highly possible that these forms of cell death are similar to TRIAD from the aspect of the extreme ER expansion. Moreover, achromasia is found in Alzheimer’s disease, motor neuron disease and Creutzfeldt-Jacob disease [6, 14].

Assuming that this type of cell death in multiple neurodegenerative diseases and TRIAD are identical, we might be able to unite cell deaths in various neurodegenerative diseases to a single prototype, and the model might enable us to generally discuss cell death of neurodegenerative diseases. The hypothesis should be further tested in the future in other neurodegenerative diseases than ALS and HD that we have analyzed.

Finally, considering with such evidences of TRIAD in HD (this study) and ALS [12], application of anti-Hippo pathway drugs such as S1P agonists should be considered positively to clinical trials against these diseases.

## Conclusions

In this study, we examined whether TRIAD, a new type of necrosis dependent on YAP and Hippo pathway, occurs in human HD brains. Our results showed activation of LATS1, suppression of Plk1, and decrease of YAP/YAPdeltaC. EM analysis also revealed typical morphological features of TRIAD. These data collectively supported that TRIAD occurs in human HD brains in vivo. In addition, biochemical and EM analyses revealed the chronological shift from early-phase to late-phase TRIAD changes, supporting the existence of TRIAD in the HD pathology in vivo.

## Additional files

Additional file 1: Figure S1. Ultrastructural analysis of additional human HD patients. ER indicates extremely expanded ER that is very homologous to the previously described ballooning of ER in TRIAD [8]. (TIFF 1777 kb)
Additional file 2: Figure S2. Ultrastructural analysis of human PSP patients. ER indicates expanded ER. (TIFF 4677 kb)
Additional file 3: Figure S3. Chronological relationship of biochemical and morphological changes in mouse HD model and human HD brains. (TIFF 784 kb)
Additional file 4: Table S1. Summary of results from HD and PSP patients. (TIFF 943 kb)
