# Supplementary figures and images for: A novel form of necrosis, TRIAD, occurs in human Huntington’s disease
Source: Acta Neuropathol Commun. 2017 Mar 8;5:19. doi: 10.1186/s40478-017-0420-1 (PMC5341362; doi:10.1186/s40478-017-0420-1)

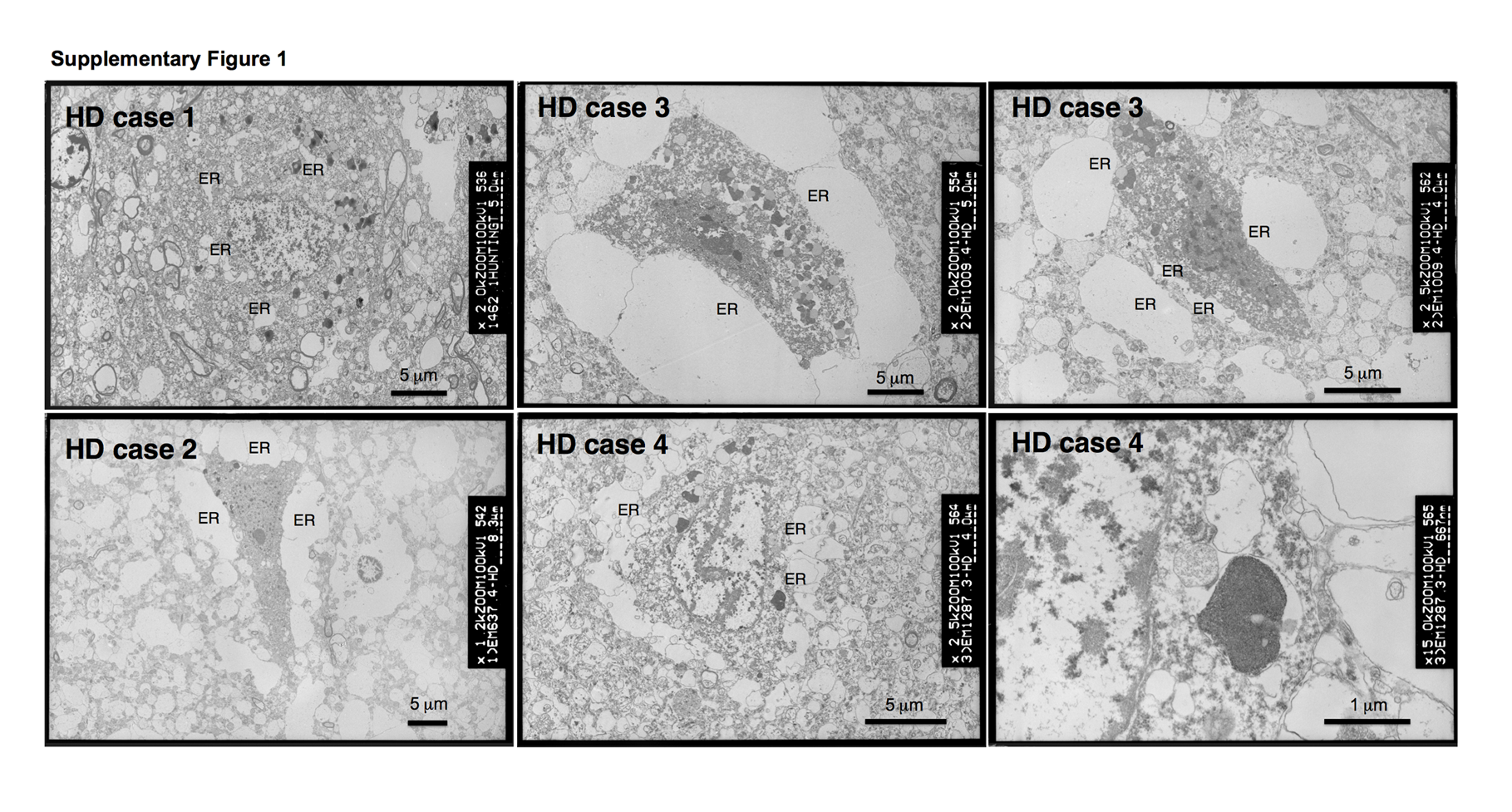

Supplement: Additional file 1: Figure S1. — Ultrastructural analysis of additional human HD patients. ER indicates extremely expanded ER that is very homologous to the previously described ballooning of ER in TRIAD [8]. (TIFF 1777 kb) [file 40478_2017_420_MOESM1_ESM.tiff]

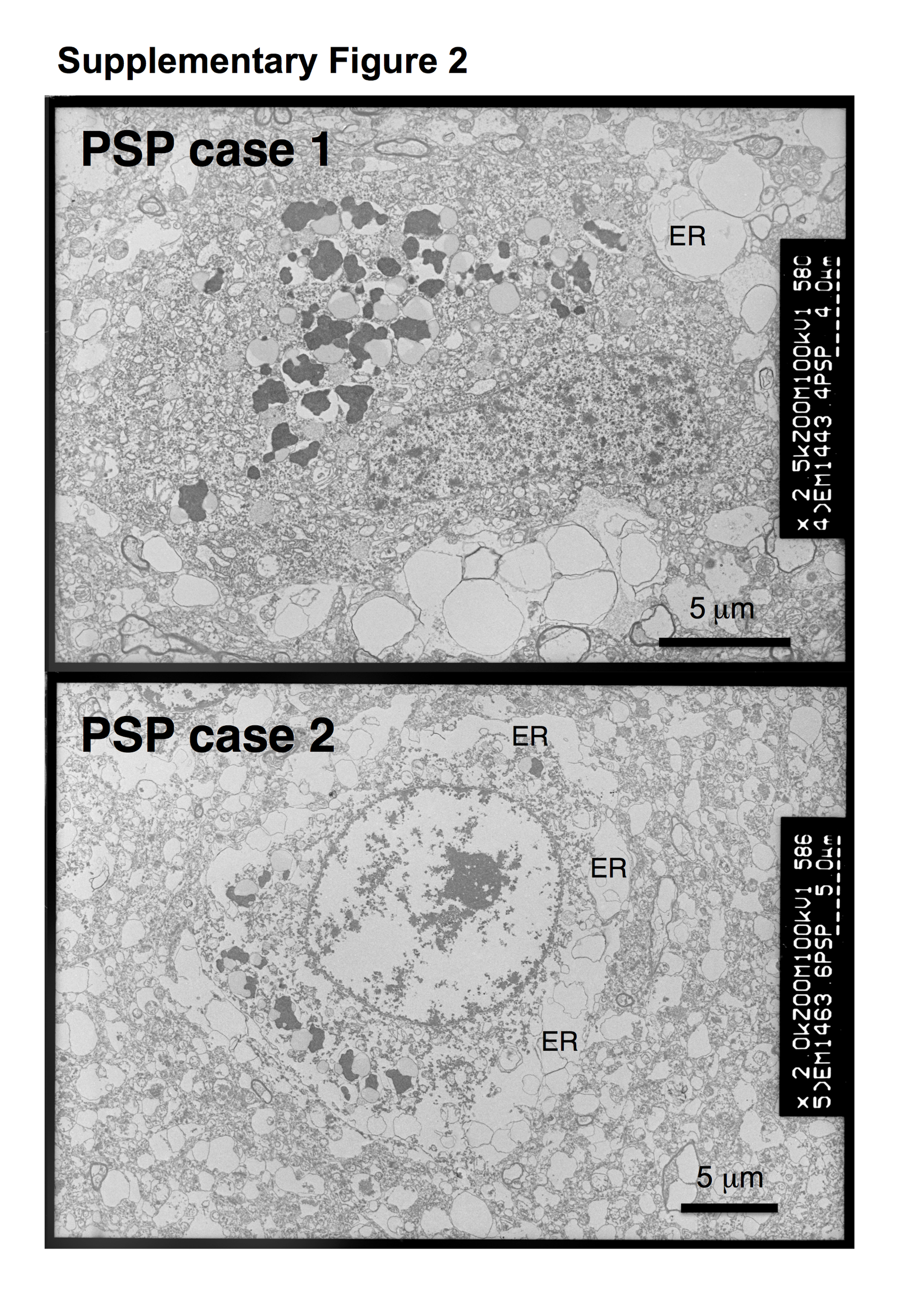

Supplement: Additional file 2: Figure S2. — Ultrastructural analysis of human PSP patients. ER indicates expanded ER. (TIFF 4677 kb) [file 40478_2017_420_MOESM2_ESM.tiff]

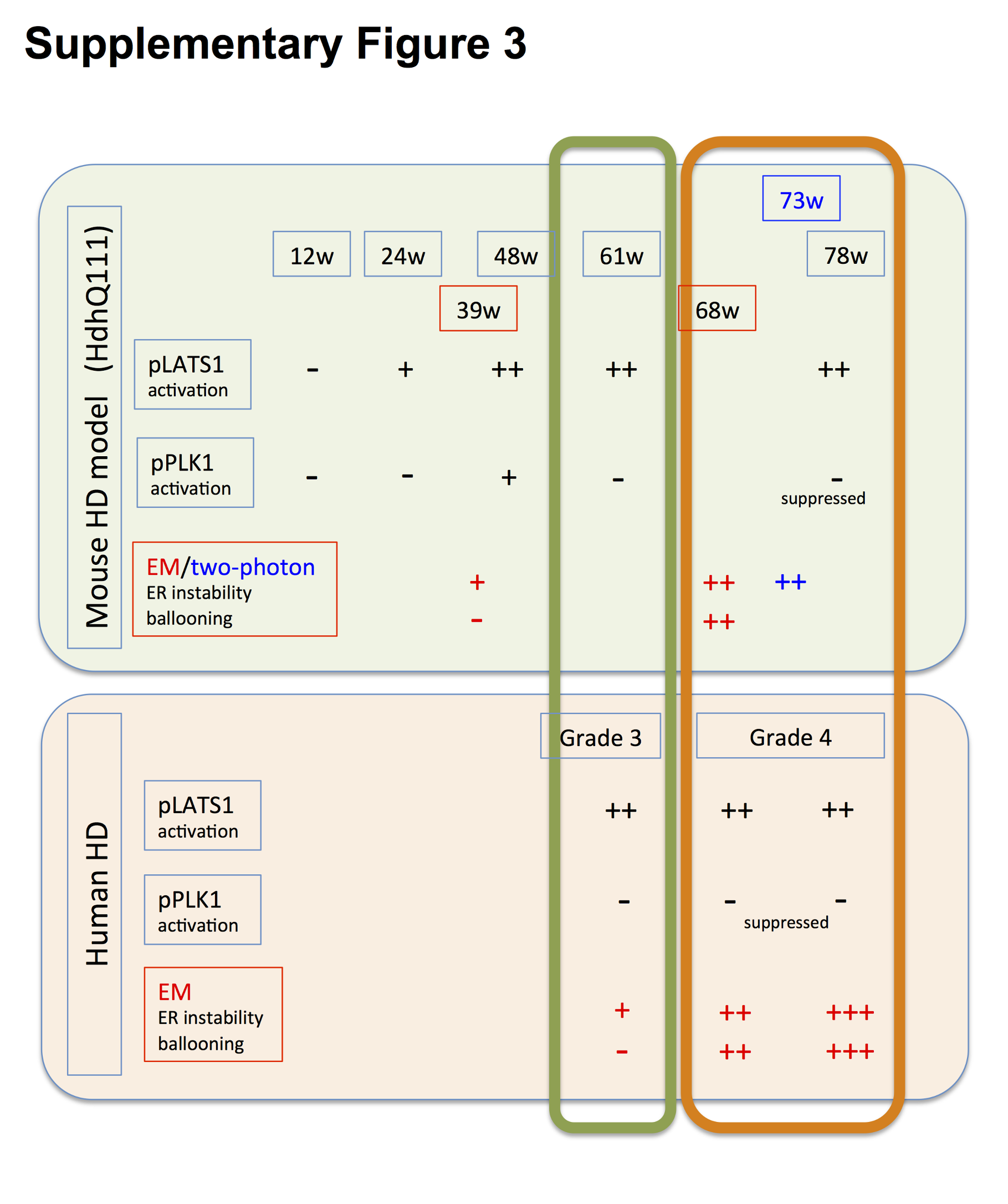

Supplement: Additional file 3: Figure S3. — Chronological relationship of biochemical and morphological changes in mouse HD model and human HD brains. (TIFF 784 kb) [file 40478_2017_420_MOESM3_ESM.tiff]

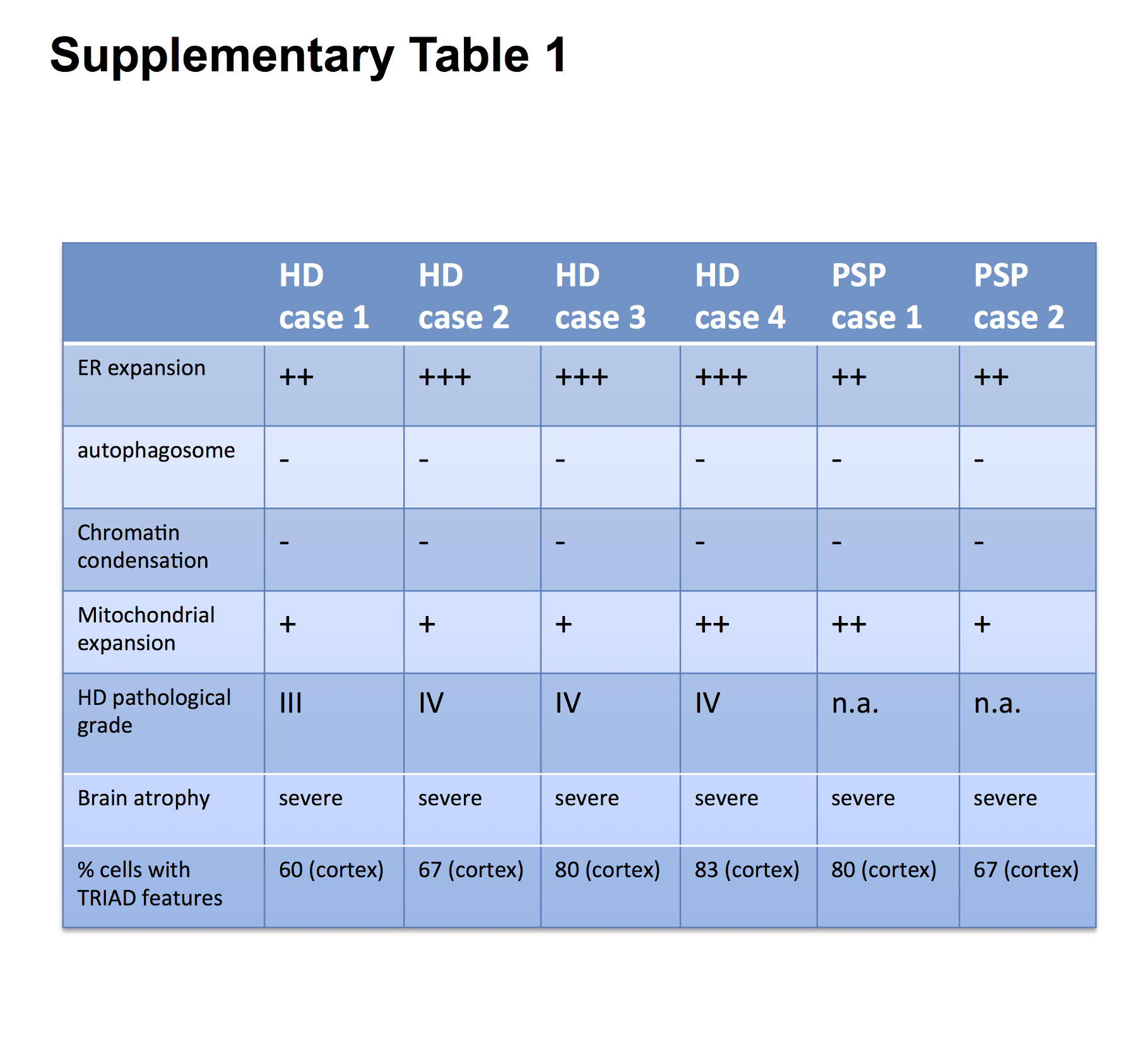

Supplement: Additional file 4: Table S1. — Summary of results from HD and PSP patients. (TIFF 943 kb) [file 40478_2017_420_MOESM4_ESM.tiff]
